# Essentiality and Transcriptome-Enriched Pathway Scores Predict Drug-Combination Synergy

**DOI:** 10.3390/biology9090278

**Published:** 2020-09-07

**Authors:** Jin Li, Yang Huo, Xue Wu, Enze Liu, Zhi Zeng, Zhen Tian, Kunjie Fan, Daniel Stover, Lijun Cheng, Lang Li

**Affiliations:** 1 Department of Biomedical Informatics, The Ohio State University, Columbus, OH 43202, USA; jin.li@osumc.edu (J.L.); xue.wu@osumc.edu (X.W.); zhizeng@whu.edu.cn (Z.Z.); Zhen.Tian@osumc.edu (Z.T.); Kunjie.Fan@osumc.edu (K.F.); Lijun.Cheng@osumc.edu (L.C.); 2 School of Informatics and Computing, Indiana University, Indianapolis, IN 46202, USA; yanghuo@iu.edu (Y.H.); enzeliu@iu.edu (E.L.); 3 Division of Medical Oncology, Department of Medicine, The Ohio State University, Columbus, OH 43202, USA; Daniel.Stover@osumc.edu

**Keywords:** drug-combination synergy prediction, drug target, gene essentiality, gene expression, KEGG pathway

## Abstract

In the prediction of the synergy of drug combinations, systems pharmacology models expand the scope of experiment screening and overcome the limitations of current computational models posed by their lack of mechanical interpretation and integration of gene essentiality. We therefore investigated the synergy of drug combinations for cancer therapies utilizing records in NCI ALMANAC, and we employed logistic regression to test the statistical significance of gene and pathway features in that interaction. We trained our predictive models using 43 NCI-60 cell lines, 165 KEGG pathways, and 114 drug pairs. Scores of drug-combination synergies showed a stronger correlation with pathway than gene features in overall trend analysis and a significant association with both genes and pathways in genome-wide association analyses. However, we observed little overlap of significant gene expressions and essentialities and no significant evidence that associated target and non-target genes and their pathways. We were able to validate four drug-combination pathways between two drug combinations, Nelarabine-Exemestane and Docetaxel-Vermurafenib, and two signaling pathways, PI3K-AKT and AMPK, in 16 cell lines. In conclusion, pathways significantly outperformed genes in predicting drug-combination synergy, and because they have very different mechanisms, gene expression and essentiality should be considered in combination rather than individually to improve this prediction.

## 1. Introduction

High-throughput genomic analyses are changing the landscape of cancer diagnosis [1,2,3,4,5,6,7]. A recent clinical study at Johns Hopkins University [8] indicated genomic changes in 25%–98% of cancer patients that could be targeted using drugs approved by the US Federal Drug Administration (FDA) and under clinical trial investigation, with the report noting a median of four actionable genetic alterations. These striking results imply the need to consider multi-drug therapies in most cancer patients, and investigations such as the Personalized Oncology Study at the University of Michigan [7] and the Pediatric Cancer Precision Medicine Study at Indiana University [9] are evaluating recommendations for multi-drug and -target interventions.

The steady growth of research and development of multi-drug therapies during the last decade highlights the profound understanding of the cancer research and drug development communities of the complexity of cancer biology and disease. The number of FDA-approved multi-drug cancer therapies increased from one combination in 2007 to 15 in 2018, and data reported in https://clinicaltrials.gov/ reflect an increase in clinical studies of multi-drug cancer therapies for the same period‒from 445 in 2007 to 798 in 2018. We have observed a similar trend in the pre-clinical cancer research community. PubMed data demonstrate an increase in the publication of in vitro or animal studies of multi-drug therapies for cancer from 12,341 in 2001 to 26,323 in 2018.

A drug combination effect (inhibition) on the cell viability can be either greater or lesser than their additive effect achieved by their individual use, resulting in synergistic or antagonistic interactions [10]. Those drug combinations working synergistically require much lower doses of each drug to achieve the same effect on cell viability and are therefore much more appealing for clinical use.

High-throughput preclinical approaches that include both drug-screening experiments and computational biology approaches are crucial to identify synergistic drug combinations. An exponentially growing number of combination drugs, heterogeneous disease mechanisms, and cell cultural models challenge the capacity of experimentation to assess drugs [11], so rapid and efficacious computational approaches could play a critical complementary role in their evaluation [12].

Sources of data in the public domain regarding large-scale screenings of drug combinations include those of the National Cancer Institute (NCI), Merck & Co., Inc., and AstraZeneca in partnership with the Sanger Institute. The NCI ALMANAC (A Large Matrix of Anti-Neoplastic Agent Combinations) database comprises more than 5000 drug pairs that utilize 104 drugs in 60 NCI cancer cell lines [13], while the Merck & Co. dataset screens 583 drug combinations in 39 cancer cell lines [11], and the drug-combination dataset compiled by AstraZeneca/Sanger for the DREAM (Dialog for Reverse Engineering Assessments and Methods) Challenge comprises 910 combinations across 85 cancer cell lines, but it does not include drug names [14].

Computational approaches to model and predict the synergy of drug combinations include machine-learning methods (DeepSynergy [15]; random forest (RF); extreme gradient boosting (XGBoost) [16]; and graph convolutional network (GCN) [17]), network methods [18,19], and systems biology methods [10,20,21]. Though these computational biology models differ in their analytical and theoretical methods, they share similar feature sets, including drug and cell-line features. Drug features include target genes [22,23], American Therapeutic Chemical Classification (ATC) codes [23,24], chemical structures [15,22,24,25], drug responses [26], and side effects and off side effects [25]. Transcriptome [15,24,26] is the most popular among cell-line features, which also includes transcriptome-enriched pathways [23,25,26,27], gene ontology, and protein‒protein interaction [24].

Several important unanswered biology questions remain regarding the prediction of drug-combination synergy. Are pathways more informative than individual genes in predicting drug synergies? Does gene essentiality, measured by CRISPR (clustered regular interspaced short palindromic repeats) or shRNA (short hairpin RNA), provide the same drug synergy prediction as gene expression? Do data regarding the expression or essentiality of drug target genes predict drug synergy? Most current computational biology approaches focus on optimizing prediction performance, but none were designed to answer these questions.

To investigate these questions, we applied our proposed systems pharmacology models, and statistical analyses. Firstly, we used drug combination synergy data from NCI ALMANAC, and employed logistic regression to test the statistical significance of gene and pathway features in that interaction. Gene expression, gene essentiality, KEGG pathways, and drug targets were used in the analysis. Then, we compared the pathways and genes, gene expression and essentiality, targets and non-targets in drug combination synergy prediction. At last, a validation using gene expression in 16 cell lines was performed.

## 2. Materials and Methods

### 2.1. Data Source

#### 2.1.1. Data Regarding the Synergy of Drug Combinations

We employed data accumulated in the NCI ALMANAC database regarding the synergy of drug combinations [13]. The ALMANAC project involved the systematic evaluation of the therapeutic activity of over 5000 pairs of 104 drugs approved by the FDA against a panel of 60 well-characterized human tumor cell lines (NCI-60). We employed a curated drug-target dataset to exclude drugs without targets [28,29] and finally included 2243 combinations of 69 drugs with targets in our analysis.

We evaluated the potential synergistic drug combinations utilizing ComboScores, as defined by Holbeck’s group, to grade the level of presence or absence synergy of each drug in a given combination. Holbeck’s group defined the Drug ComboScore [13] as a modification of Bliss independence. Let $Y^{A_{p}B_{q}}$ be the growth fraction for a cell line exposed to the pth concentration of Drug *A* and the qth concentration of Drug *B*, defined as:

(1) $$Y^{A_{p}B_{q}}=100*\frac{T_{1}^{A_{p}B_{q}}-T_{0}}{T_{1}^{0}-T_{0}}$$

where $T_{0}$ is the time zero measurement, $T_{1}^{A_{p}B_{q}}$ is the endpoint measurement after 2 days under both drugs *A* and *B*, and $T_{1}^{0}$ is the endpoint measurement after 2 days for the control well. Define $Y^{A_{p}}$, $Y^{B_{q}}$ as the growth fractions when exposed to either Drug *A* or Drug *B* alone. The expected growth fraction for the combination is:

(2) $$Z^{A_{p}B_{q}}=\{\begin{array}{cc} \min\left(Y^{A_{p}},\;Y^{B_{q}}\right) & Y^{A_{p}}\leq 0\;\mathrm{or}\;Y^{B_{q}}\leq 0\\ \frac{1}{100}\left({\tilde{Y}}^{A_{p}}*{\tilde{Y}}^{B_{q}}\right) & otherwise \end{array}$$

where $\tilde{Y}=\min\left(Y,100\right)$ truncates the growth fraction at 100. The final ComboScore for the cell line and the drug combination is the mean of the differences in expected versus observed growth fractions:

(3) $$Y^{AB}=\frac{1}{n}\underset{p,q}{\sum }Y^{A_{p}B_{q}}-Z^{A_{p}B_{q}}$$

where *n* is the number of the combinations for drugs *A* and *B* under different doses. The ComboScore ranges from −100 to 100.

We calculated a binary synergy status $S^{AB}$ for drugs *A* and *B* based on a ComboScore with a threshold of 10, above which a drug combination is considered synergistic.

(4) $$S^{AB}=\{\begin{array}{cc} 1 & Y^{AB}>10\\ 0 & Y^{AB}\leq 10 \end{array}$$

#### 2.1.2. Transcriptome Data

We downloaded the baseline transcriptome data for the 60 NCI cancer cell lines from NCI’s CellMiner^™^ database (Genomics and Pharmacology Facility, Developmental Therapeutics Branch, Center for Cancer Research, NCI) but excluded one cell line, MDA_N, for poor quality control [30]. This yielded 59 cell-line transcriptomes, which we analyzed using the Affymetrix^®®^ Human Genome U133 (HG-U133) Plus 2.0 platform [31]. We employed the R package “affy” to process CEL files, which made up the raw microarray data. MAS5.0 was used to normalize data, and the probes were matched to gene symbols. We calculated information regarding the presence, marginality, or absence of probe activity (PMA; present/margin/absent) in a sample for each probe and transformed the data to ascertain the transcriptome status. We considered a gene active when it demonstrated any probe activity and inactive when probe activity was absent. We used normalized gene expression in the analysis.

#### 2.1.3. Essentiality Data

Gene essentiality was assessed by shRNA screening. We downloaded shRNA data from the Dependency Map (DepMap) portal of the Broad Institute [32], performed a genome-wide pooled loss-of-function screening among cancer cell lines across approximately 100 k shRNAs, and applied the DEMETER2 (D2) analytical framework to this RNAi screening dataset [33]. We normalized the gene dependency score, D2, such that the median of the average score across cell lines was −1 for reference essential gene sets and zero for the control gene sets [34]. A threshold score of −0.5 was used to distinguish essential (below −0.5) and nonessential (above −0.5) genes.

#### 2.1.4. Training and Validation Data

Training data included 43 cancer cell lines with both base-line transcriptome and essentiality data as features. Validation samples were 16 NCI cancer cell lines with only transcriptome data.

Pathway analysis utilized data regarding transcriptome, essentiality, and pathway features as enumerated by the Kyoto Encyclopedia of Genes and Genomes (KEGG) [35]. We employed the R package “KEGGREST” to download the genes from KEGG.

### 2.2. Features

We applied several rules to filter genes for analysis. Genes were excluded that were active (present) in fewer than 20% (8.6) of cell lines, demonstrated a coefficient of variance (CV) below 0.1, or for the essential data, were not essential in all 43 cell lines. After filtering, 3024 gene expressions and 4381 gene essentialities remained, and their scores served as gene features

In constructing pathway features, we added drug target information into the calculation, utilizing target interactions reported by Feixiong Cheng and associates [28,29] that we acquired from the DrugBank [36], Therapeutic Target (TTD) [37], and PharmGKB [38] databases. The following analysis included calculations of pathway features for 165 pathways with drug target genes. The model included two types of features. The first was based on drug targets in different cell lines, e.g., the numbers of active or essential genes in a cell line, numbers of all or common targets of drug combinations, and overlapping numbers of or proportions between active and essential genes and drug targets. The second feature type was based on cell lines and KEGG pathways, such as the numbers or proportions of active and essential genes in KEGG pathways for each cell line. Table 1 details these features.

### 2.3. Logistic Regression Models in Selecting Features for Drug-Synergy Prediction

In gene analysis, we built a logistic regression model using the score of expression or essentiality for each gene as the given feature.

In analyzing and selecting features to predict drug synergy in pathway analysis, we trained logistic models with different types of features and performed three groups of analysis. Pathway Analysis 1 utilized the first type of feature only, Pathway Analysis 2, the second type only, and Pathway Analysis 3, all features.

For each gene or pathway, we chose the minimum *p*-value among all the drug combinations to represent the overall *p*-value for the given gene or pathway. In the gene-level modes, we set a genome-wide threshold for Bonferroni correction of multiple comparisons $10^{-5}$ based on expression of 3024 genes and essentiality of 4381 ($10^{-5}\approx 0.05/3024\approx 0.05/4381$). In the pathway analysis, we set the Bonferroni correction threshold $2.66*10^{-5}$ based on 165 pathways and 114 drug combinations ($2.66*10^{-5}=\frac{0.05}{165*114}$).

To assess and compare the models, we calculated multiple levels of the false discovery rate (FDR). For a given *p*-value threshold, FDR is calculated as the ratio of the number of expected to the number of observed significant results.

### 2.4. Model Training and Validation

Training and validation focused on the transcriptome features and their related pathway features in predicting drug-combination synergy. Unfortunately, no additional data were available to validate gene essentiality features and their pathway features. 

## 3. Results

It is very challenging to construct a model to predict the synergy of drug combinations that show synergy in only a very few cell lines, so we examined 114 drug combinations that showed synergy in at least five cell lines.

### 3.1. In Overall Trend Analysis, Pathway Features Showed Stronger Statistical Correlation Evidence Than Gene Features with Drug-Combination Synergy Scores

**Figure 1:**
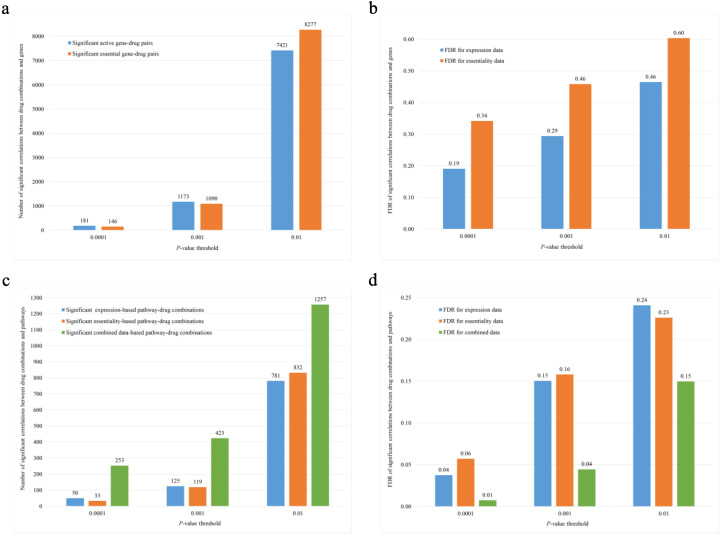
Comparison of significant correlations between drug pairs and genes and drug pairs and pathways under different thresholds. (**a**,**b**) Number and false discovery rate (FDR) of significant correlation between drug pairs and genes. (**c**,**d**) Number and FDR of significant correlation between drug pairs and pathways.

When we used the same *p*-value thresholds, 0.01, 0.001, 0.0001, the FDRs of gene expression and essentiality features varied from 0.19 to 0.60, and gene expression features were somewhat better predictors of drug synergy than essentiality features with lower FDRs (Figure 1a,b). However, pathway analysis using the same *p*-value thresholds demonstrated uniformly lower FDRs of pathway than gene features, varying between 0.01 and 0.24. Gene expressions and essentialities did not differ among pathways. In particular, we observed lower FDRs of the pathway features that integrated data of both expression and essentiality than those based on either expression or essentiality alone (Table 2, Figure 1c,d).

### 3.2. Genome-Wide Association Analyses Revealed Significant Associations of Genes and Pathways with Scores of Drug Synergy

**Figure 2:**
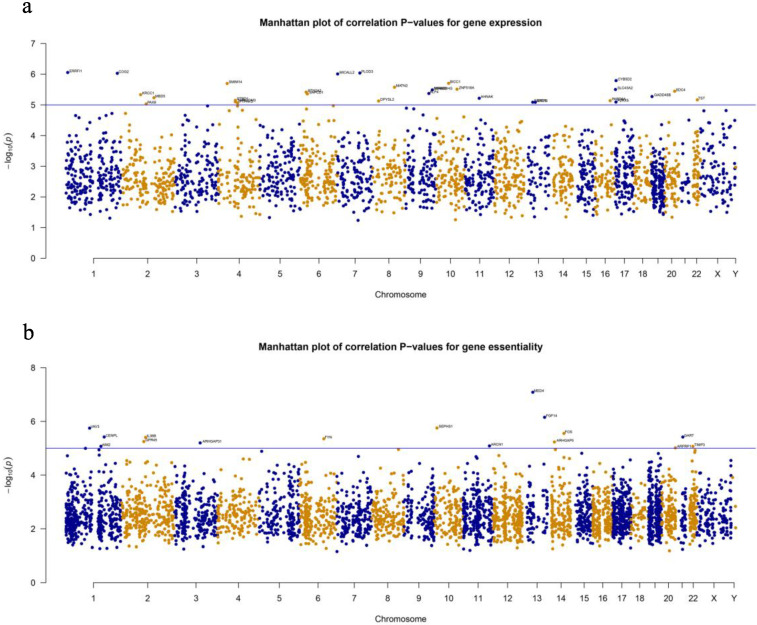
Manhattan plots of correlation *p*-values. (**a**) Gene expression data, (**b**) gene essentiality data. In these figures, the X axis represents the genes, and the Y axis represents the overall *p*-value (–log10).

Gene analyses demonstrated significant association of 30 expressed genes and 16 essential genes with drug synergy scores under the Bonferroni threshold, but they shared no common gene. Supplementary Table S1 delineates these genes, and they are marked in Figure 2. In these figures, the X axis represents the genes arranged from chromosomes, and the Y axis, the overall *p*-value (−log10).

**Figure 3:**
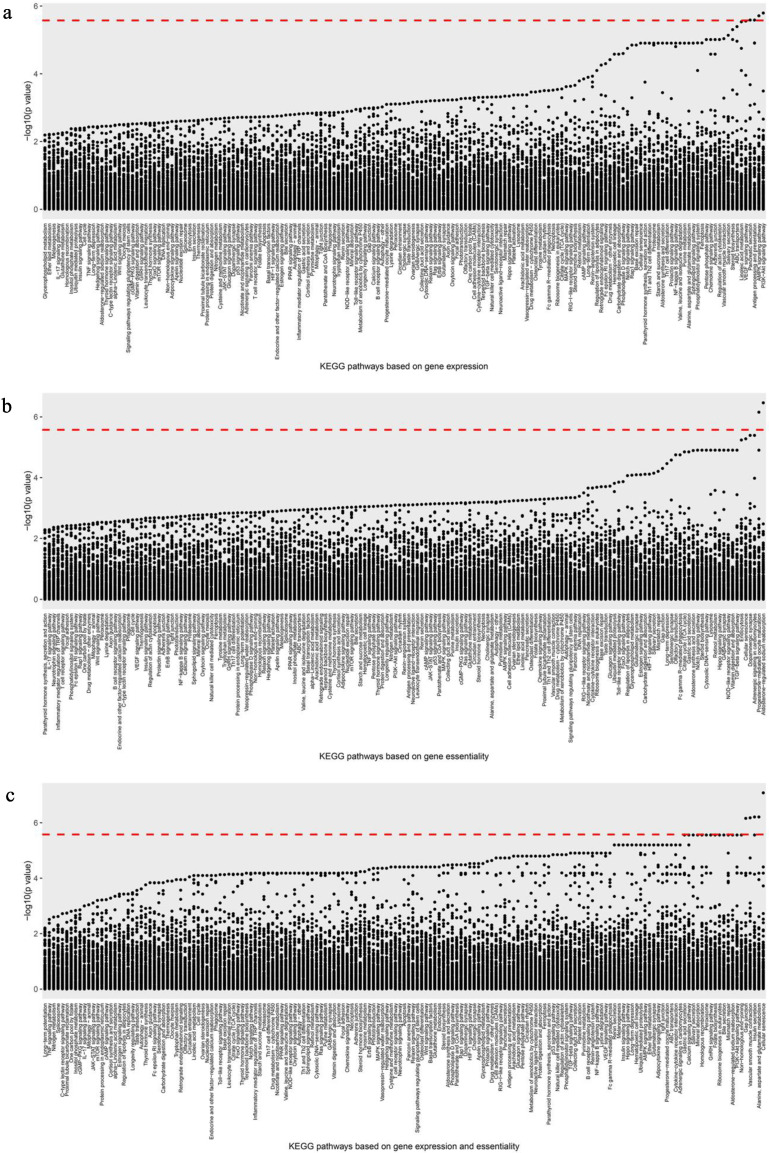
Scatter plots of correlation *p*-values in Pathway Analysis 3. (**a**) Gene expression data, (**b**) gene essentiality data, (**c**) combined data. In these figures, the X axis represents the pathways, and the Y axis represents the overall *p*-value (−log10).

For drug-synergy scores under the Bonferroni threshold, Pathway Analysis 3 demonstrated significant association of four pathways using gene expression (PI3K-AKT and AMPK signaling pathways, antigen processing and presentation, and pancreatic secretion), two using essentiality (aldosterone-regulated sodium reabsorption and progesterone-mediated oocyte maturation), and five using the combined data (cellular senescence, alanine, aspartate and glutamate metabolism, insulin secretion, vascular smooth muscle contraction, and gap junction). However, there was no common pathway among them. Figure 3 shows the distribution.

Under a genome-wide adjusted significance level, these association analyses revealed significant correlation between drug-synergy scores and several genes and pathways. However, the different genes and pathways between expression and essentiality indicated different mechanisms of gene expression and essentiality at work in the synergistic activity of the drug combinations.

### 3.3. Overlap Was Limited between Significant Gene Expressions and Essentialities in Predicting Drug Synergy

**Figure 4:**
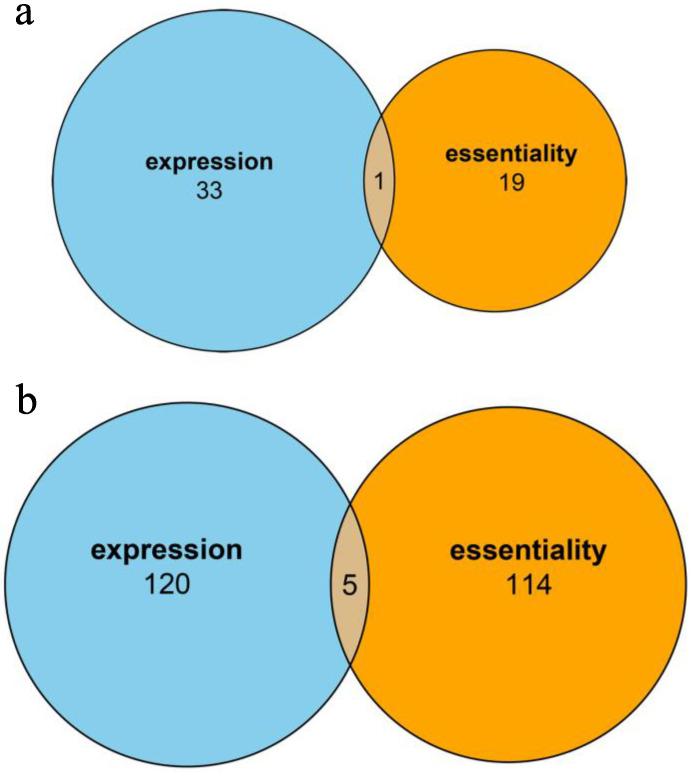
Venn plots illustrating results between gene expression and essentiality. (**a**) Number of significant drug combination-gene relationships using gene expression and essentiality, (**b**) number of significant drug combination-pathway relationships using gene expression and essentiality.

Gene analysis revealed 473 common genes among expression (3024 genes) and essentiality (4381 genes) datasets. Under an overall *p*-value threshold of 0.001, drug combinations correlated significantly with expression in 34 genes and essentiality in 20, with only one gene showing overlap in expression and essentiality (Figure 4a).

Under an overall *p*-value threshold of 0.001, Pathway Analysis 3 demonstrated significant correlation of drug combinations and expression in 125 genes and essentiality in 119, with only five genes showing overlap in expression and essentiality (Figure 4b).

Both the gene and pathway analyses revealed quite different information regarding gene expression and essentiality for predicting drug-combination synergy.

### 3.4. No Statistically Significant Evidence Supported Relationship between Target and Non-Target Genes and Pathways in the Prediction of Drug Synergy

**Figure 5:**
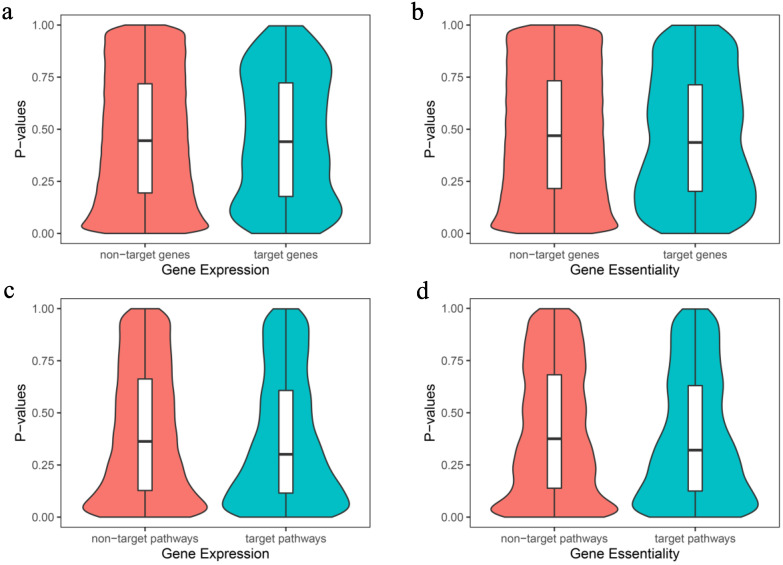
Violin plots of *p*-values for target and non-target genes (**a**,**b**) and pathways (**c**,**d**) using (**a**) gene expression, (**b**) gene essentiality, (**c**) gene expression, and (**d**) gene essentiality.

For each drug combination, we separated gene features into target and non-target features, including expression and essentiality features, and we further differentiated whether target genes were in a pathway, including pathways defined by gene expressions and essentialities. Comparison between the target and non-target groups showed no significant differences between genes (Figure 5a,b) or pathways (Figure 5c,d). In the violin plot, there is a box in each violin. The lines in the box are first quartile (Q_1_), medium (Q_2_), and third quartile (Q_3_). The curves of violin plots also show the probability density of the data at different values, smoothed by a kernel density estimator.

### 3.5. Feature Comparison in Pathway Analysis

Pathway Analysis 1 included information regarding drug-combination targets and gene expression and essentiality based on cell-line information and did not include pathway information. For each drug combination, a separate model was constructed for gene expression and for essentiality. Pathway Analyses 2 and 3 included pathway information, so for each drug combination and pathway, a separate model was trained for gene expression and for essentiality. For each drug combination, we set the minimum *p*-value among all pathways as the overall *p*-value for the drug combination to allow comparison with the model constructed based on the first group of features. Table 2 shows the FDRs using different thresholds.

We first compared the drug combination level results. In Pathway Analysis 1, of 114 drug combinations, zero (expression data), one (essentiality data), and one (combined data) FDR = (NA, 0.11 and 0.11) can be properly predicted under a *p*-value threshold of 0.001.

In Pathway Analysis 2, 25 (expression data), 15 (essentiality data), and 35 (combined data) FDR = (4.4 × 10^−3^, 7.33 × 10^−3^ and 3.14 × 10^−3^) can be properly predicted.

In Pathway Analysis 3, 36 (expression data), 26 (essentiality data), and 62 (combined data) FDR = (3.06 × 10^−3^, 4.23 × 10^−3^ and 1.77 × 10^−3^) can be properly predicted. Obviously, pathway features were more informative than the basic cell-line information, and integrated features in Pathway Analysis 3 performed best.

We then compared the results regarding drug combinations and pathway level between Pathway Analyses 2 and 3. In Pathway Analysis 2, 43 (expression data), 25 (essentiality data), and 78 (combined data) of drug combination-pathways FDR = (0.44, 0.75 and 0.24) can be properly predicted under a *p*-value threshold of 0.001. In Pathway Analysis 3, the numbers were 125 (expression data), 119 (essentiality data), and 423 (combined data) FDR = (0.15, 0.16 and 0.044). The integration of information regarding pathways and cell lines in Pathway Analysis 3 was more informative than the pathway information alone in Pathway Analysis 2. Results were similar using *p*-value thresholds of 0. 01 and 0.0001 (Table 2).

**Figure 6:**
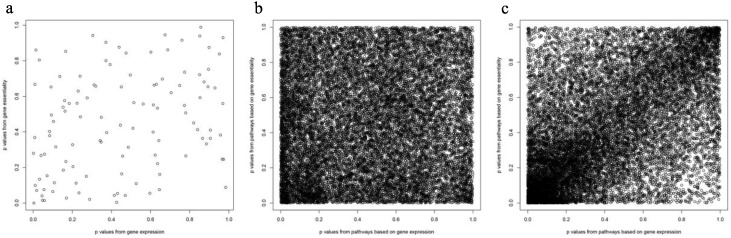
Scatter plot of the *p*-values from gene expression and essentiality. The more points in the main diagonal, the more correlation between the 2 groups. (**a**–**c**) Feature groups 1–3.

FDR results were similar or slightly better based on expression rather than essentiality and were best when expression and essentiality were combined. Subsequent analysis of the correlation between expression and essentiality to investigate the amount of information they shared yielded: Pearson (Spearman) correlation coefficients of *p*-values of 0.3105 for expression and 0.3074 for essentiality in Pathway Analysis 1; 0.0430 for expression and 0.0453 for essentiality in Pathway Analysis 2; and 0.5578 for expression and 0.5766 for essentiality in Pathway Analysis 3 (Figure 6). These values reflected little common information between gene expression and essentiality if they were used separately (Pathway Analysis 2).

### 3.6. Model Validation

For validation, we selected the two most significant correlations of drug-combination pathways using gene expression data in Feature Group 3. The signaling pathways were PI3K-Akt for the combination of drugs Nelarabine and Exemestane and AMPK for the combination of Docetaxel and Vemurafenib. PI3K-Akt pathway is an intracellular signal transduction pathway that promotes metabolism, proliferation, cell survival, growth and angiogenesis in response to extracellular signals. This is mediated through serine and/or threonine phosphorylation of a range of downstream substrates. Key proteins involved are phosphatidylinositol 3-kinase (PI3K) and Akt/Protein Kinase B [39]. Nelarabine is a chemotherapy drug used in T-cell acute lymphoblastic leukemia. Its target gene is POLA1. Exemestane is a member of the class of antiestrogens known as aromatase inhibitors, which is used to treat breast cancer. Its target genes are NFE2L2 and AR. One of the central regulators of cellular and organismal metabolism in eukaryotes is AMP-activated protein kinase (AMPK), which is activated when intracellular ATP production decreases. AMPK has critical roles in regulating growth and reprogramming metabolism, and has recently been connected to cellular processes such as autophagy and cell polarity. The AMPK signaling pathway coordinates cell growth, autophagy, and metabolism [40]. Docetaxel is a chemotherapy medication used to treat a number of types of cancer, including breast cancer, head and neck cancer, stomach cancer, prostate cancer and non-small-cell lung cancer. Its target genes are MAP2, NR1I2, BCL2, TUBB1, MAPT, and MAP4. Vemurafenib is an inhibitor of the B-Raf enzyme for the treatment of late stage melanoma. Vemurafenib selectively binds to the ATP-binding site of BRAF (V600E) kinase and inhibits its activity, which may result in an inhibition of an over-activated MAPK signaling pathway downstream in BRAF (V600E) kinase-expressing tumor cells and a reduction in tumor cell proliferation. Its target genes are YES1, ARAF, BRAF, KDR and RAF1.

**Figure 7:**
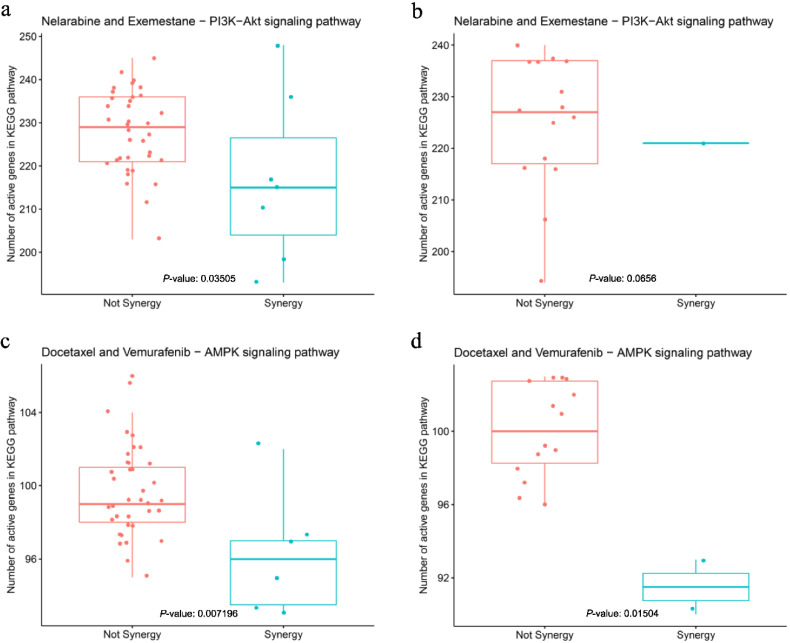
Box plot of the number of active genes in the KEGG pathway. The lines in the box are First quartile (Q1), Medium (Q2), and Third quartile (Q3). (**a**,**c**) training data; (**b**,**d**) validation data.

A non-parametric Wilcoxon test was used to compare the numbers of active genes in the pathways to classify the groups with and without synergy. The *p*-values are 0.03505, 0.0656, 0.007196 and 0.01504 for groups a–d in Figure 7. In the 16 cell lines used for validation, only one reflected synergistic activity for Nelarabine and Exemestane and only two for Docetaxel and Vemurafenib. In this small and unbalanced data, especially the validation data, these small *p*-values of significance showed significant separation of these groups in both the training and validation datasets, even when only a single feature was considered (e.g., number of active genes in the pathway) (Figure 7). The results thus proved the efficiency of the pathway features used in the models.

## 4. Discussion

In AstraZeneca’s drug-combination prediction DREAM Challenge [14], the authors summarized and compared different models and features and observed better prediction performance when such data as KEGG information was added to the base-line model. Similarly, our model employed a pathway containing several genes rather than a single gene because we believed their regulatory relationship would hold some special function and make more biological meaning. More powerful prediction of drug synergy has been proven utilizing pathways rather than either expression or essentiality of a single gene. In this paper, in order to directly comparing genes and pathways, only 1 pathway was used in each model. The pathway interactions or crosstalk were not considered in the analysis.

There is no statistically significant evidence between target and non-target genes/pathways in the prediction of drug synergy. The possible reason is that the drug target information is still limited. Even several databases are used to acquire the drug targets, it is still limited and unbalanced. The number of target genes varies among drug types, such as monoclonal antibodies, Alkylating agents, Anthracyclines, and Mitotic inhibitors. Therefore, there are some biases on target-based pathways.

**Figure 8:**
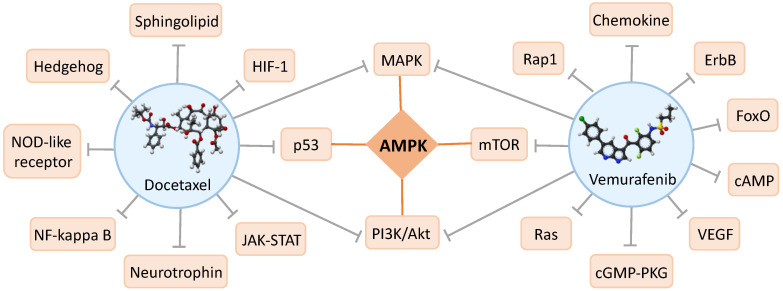
Docetaxel, Vemurafenib and targeted pathways.

Currently, NCI ALMANAC is the largest drug combination synergy data. However, there are only 60 cell lines, and only 16 cell lines in the validation. This small sample size limited the power and robustness of our model. The significant correlations of AMPK signaling pathway and the drug combination Docetaxel and Vemurafenib were further validated in literature. The synergistic effect of this drug combination has been confirmed by in vitro study [41]. The targeted pathways were mapped based on the drug targets. Both of these two drugs target several pathways, and they target two common pathways (PI3K/Akt signaling pathway and MAPK signaling pathway), but none of them target AMPK signaling pathway directly. The interaction or crosstalk network between these pathways were further investigated. AMPK signaling is inhibited by hyperactive MAPK signaling in cancers, and the AMPK signaling can regulate MAPK signaling reversely [42]. There are crosstalk between the AMPK and PI3K/Akt pathways in breast cancer cells [43]. AMPK exerts dual effects on the PI3K/Akt pathway and mTOR pathway, stimulating PI3K/Akt and inhibiting mTOR/S6K [44,45]. AMPK Induces p53 pathway by Phosphorylating MDMX and inhibiting its activity [46] (Figure 8). There are lots of interactions or crosstalk between AMPK pathways and these directly targeted pathways, even it is not directly targeted. Therefore, it is not necessary that the most significant pathway is the directly targeted pathway, and a pathway which has interactions or crosstalk with the directly targeted pathways may also play important roles in drug synergy prediction.

## 5. Conclusions

This is the first study comparing gene expression and gene essentiality for drug synergy prediction. Gene expression and essentiality each reflect different functional mechanisms, and the consideration of one or the other has been effective in the prediction of drug synergy. Certainly, their combined consideration would enhance that prediction, and prediction should improve even further as more and more gene essentiality data become publicly available.

## Figures and Tables

**Table 1:** Definitions of pathway features in the models.

Data	Feature	Feature Description	Type
Drug-combination targets	n_D_union_ab_	Number of total drug targets for Drug A or Drug B	
	n_D_intersection_ab_	Number of total drug targets for both Drug A and Drug B	
KEGG pathways	n_k_	Number of genes in KEGG Pathway K	
Cell lines	n_cell_c_expression_	Number of active genes in Cell Line C	1
	n_cell_c_essentiality_	Number of essential genes in Cell Line C	1
Drug-combination targets in cell lines based on gene expression	n_cell_c_expression_D_union_ab_	Number of total drug targets for Drug A or Drug B that are active in Cell Line C	1
	n_cell_c_expression_D_intersection_ab_	Number of total drug targets for both Drug A and Drug B that are active in Cell Line C	
	n_cell_c_expression_D_union_ab/_n_cell_c_expression_	Ratio of the number of active drug targets for Drug A or Drug B to all active genes in Cell Line C	
	n_cell_c_expression_D_intersection_ab/_n_cell_c_expression_	Ratio of the number of active drug targets for Drug A and Drug B to all active genes in Cell Line C	
Drug-combination targets and cell lines based on gene essentiality	n_cell_c_ essentiality _D_union_ab_	Number of total drug targets for Drug A or Drug B that are essential in Cell Line C	1
	n_cell_c_essentiality _D_intersection_ab_	Number of total drug targets for both Drug A and Drug B that are essential in Cell Line C	
	n_cell_c_essentiality_D_union_ab/_n_cell_c_essentiality_	Ratio of the number of essential drug targets for Drug A or Drug B relative to all essential genes in Cell Line C	
	n_cell_c_essentiality_D_intersection_ab/_n_cell_c_essentiality_	Ratio of the number of essential drug targets for Drug A and Drug B relative to all essential genes in Cell Line C	
KEGG pathways and cell lines based on gene expression	n_cell_c_expression_kegg_k_	Number of active genes in KEGG Pathway K for Cell Line C	2
	n_cell_c_expression_kegg_k/_n_cell_c_expression_	Ratio of the number of active genes in KEGG Pathway K relative to all active genes in Cell Line C	
KEGG pathways and cell lines Based on gene essentiality	n_cell_c_essentiality_kegg_k_	Number of essential genes in KEGG Pathway K for Cell Line C	2
	n_cell_c_essentiality_kegg_k/_n_cell_c_essentiality_	Ratio of the number of essential genes in KEGG Pathway K to all essential genes in Cell Line C	

KEGG, Kyoto Encyclopedia of Genes and Genomes.

**Table 2:** Results of pathway analysis.

Group		Gene Expression				Gene Essentiality				Combined Expression and Essentiality			
		Threshold	Number Observed	Number Expected	FDR	Threshold	Number Observed	Number Expected	FDR	Threshold	Number Observed	Number Expected	FDR
1		0.0001	0	0.01	NA	0.0001	1	0.01	0.01	0.0001	1	0.01	0.01
		0.001	0	0.11	NA	0.001	1	0.11	0.11	0.001	1	0.11	0.11
		0.01	4	1.14	0.29	0.01	2	1.14	0.57	0.01	7	1.14	0.16
2	DD *	0.0001	7	0.01	1.43 × 10^−3^	0.0001	2	0.01	0.005	0.0001	11	0.01	9.09 × 10^−4^
		0.001	25	0.11	4.4 × 10^−3^	0.001	15	0.11	7.33 × 10^−3^	0.001	35	0.11	3.14 × 10^−3^
		0.01	72	1.14	0.016	0.01	77	1.14	0.015	0.01	85	1.14	0.013
	DDP ^#^	0.0001	8	1.88	0.24	0.0001	3	1.88	0.63	0.0001	16	1.88	0.12
		0.001	43	18.81	0.44	0.001	25	18.81	0.75	0.001	78	18.81	0.24
		0.01	443	188.1	0.42	0.01	311	188.1	0.60	0.01	519	188.1	0.36
3	DD *	0.0001	23	0.01	4.35 × 10^−4^	0.0001	13	0.01	7.69 × 10^−4^	0.0001	37	0.01	2.72 × 10^−4^
		0.001	36	0.11	3.06 × 10^−3^	0.001	26	0.11	4.23 × 10^−3^	0.001	62	0.11	1.77 × 10^−3^
		0.01	86	1.14	0.013	0.01	61	1.14	0.019	0.01	98	1.14	0.012
	DDP ^#^	0.0001	50	1.88	0.038	0.0001	33	1.88	0.057	0.0001	253	1.88	7.43 × 10^−3^
		0.001	125	18.81	0.15	0.001	119	18.81	0.16	0.001	423	18.81	0.044
		0.01	781	188.1	0.24	0.01	832	188.1	0.23	0.01	1257	188.1	0.15

DD *, drug-pair model; the minimum *p*-value among all pathways was set to be the overall *p*-value for a drug pair. DDP ^#^, drug pair-pathway model; each *p*-value for every drug combination-pathway model was evaluated. FDR, false discovery rate. NA, not applicable.

## Supplementary Materials

The following are available online at https://www.mdpi.com/2079-7737/9/9/278/s1, Table S1: The overall *p*-values of genes based on gene expression and essentiality.
